# Mental health impacts of flooding: a controlled interrupted time series analysis of prescribing data in England

**DOI:** 10.1136/jech-2017-208899

**Published:** 2017-08-31

**Authors:** Ai Milojevic, Ben Armstrong, Paul Wilkinson

**Affiliations:** Department of Social and Environmental Health Research, London School of Hygiene and Tropical Medicine, London, UK

**Keywords:** environmental epidemiology, mental health, prescribing, primary health care, time-series

## Abstract

**Background:**

There is emerging evidence that people affected by flooding suffer adverse impacts on their mental well-being, mostly based on self-reports.

**Methods:**

We examined prescription records for drugs used in the management of common mental disorder among primary care practices located in the vicinity of recent large flood events in England, 2011–2014. A controlled interrupted time series analysis was conducted of the number of prescribing items for antidepressant drugs in the year before and after the flood onset. Pre–post changes were compared by distance of the practice from the inundated boundaries among 930 practices located within 10 km of a flood.

**Results:**

After control for deprivation and population density, there was an increase of 0.59% (95% CI 0.24 to 0.94) prescriptions in the postflood year among practices located within 1 km of a flood over and above the change observed in the furthest distance band. The increase was greater in more deprived areas.

**Conclusions:**

This study suggests an increase in prescribed antidepressant drugs in the year after flooding in primary care practices close to recent major floods in England. The degree to which the increase is actually concentrated in those flooded can only be determined by more detailed linkage studies.

## Introduction

Floods are the most common type of natural disaster globally. In the UK, around 1.8 million people live in properties with an annual risk of flooding greater than 1 in 75,^1^ a number expected to increase because of climate change and the pressures of development.^1^ Although the immediate risk of death and injury from flooding is small in high-income countries such as the UK, there is growing evidence of medium to longer term adverse effects on mental health^2–7^: increased rates of depression/anxiety and post-traumatic stress disorder as well as exacerbation of illness in persons with pre-existing depression have been reported in flood-affected populations.^4–6^ However, the evidence remains inconclusive in part because floods can only be studied retrospectively and usually without robust preflood baseline data,^8 9^ a methodological challenge that also applies to other natural and man-made disasters.^10–12^

In this study, we examine the mental health impacts of floods in England using routine prescriptions data which can provide data for both preflood and postflood periods in flooded and non-flooded populations.

## Methods

### Practices prescribing data

The General Practice Prescribing Data covers all National Health Service prescriptions written by general practitioners and non-medical prescribers (including nurses and pharmacists) attached to primary care practices in England and dispensed in the community in the UK.^13^ It records the total number of items, total net ingredient cost, actual cost and quantity prescribed and dispensed by practice, month and medicine presentation (British National Formulary code). We extracted data on prescriptions of antidepressant drugs (see eList in the online supplementary material) and other, non-antidepressant, drugs.

10.1136/jech-2017-208899.supp1 Supplementary file 1

Practices, identified by national code, were mapped using the coordinates of the centroid of the unit postcode (approximately 14 households *per* postcode) of their addresses at the time the prescriptions were dispensed. Practices located within 10 km of the flood boundaries of the target flood events described below were analysed. Practices which changed address in the interval between a year before and after the flood onset were excluded. Note that for all practices, including those located within very short distances of a flood area, only a small proportion of the practice’s registered patients would have been affected by flooding.

### Measurement of flood exposure

We used the Recorded Flood Outlines (V.201602, UK Environment Agency), a Geographic Information System database which contains the boundaries of inundated land identified from multiple sources including surveys, visual investigation and aerial photographs, together with related attributes such as start and end date of the flood event and flood type (fluvial, tidal and/or coastal). Preflood and postflood periods were defined with respect to start date of the flood. The study was confined to the large flood events, June 2011–November 2014, which each flooded more than 500 addresses (‘delivery points’ in ONS Code Point data): the 2013 North East tidal surge, the East coast tidal event, the East Midland tidal surge, the East of England tidal surge and the South West floods in winter 2013/2014 (see eFigure1 in the online supplementary material). All these areas were first inundated in December 2013 except the mostly fluvial floods of the South West, which were first flooded between December 2013 and February 2014. In total, 6000 households and 13 000 people were estimated to have been flooded by overlaying with 2011 census data.^14^

Each practice was classified by quintile of socioeconomic deprivation using the Income and Employment domains of 2015 English Index of Multiple Deprivation^15^ for the Lower-level Super Output Area (average population 1500) where the practice is located and by quintile of the averaged population density across output areas within 2 km of the practice (a radius chosen to represent the level of rurality of the main catchment area of the practice).

### Analysis

Analysis was based on a controlled interrupted time series Poisson regression^16 17^ in which monthly prescriptions for antidepressant drugs were compared in the 12 months before and after the flood onset and stratified by distance of the practice from the nearest flood boundary (using bands defined at 0, 1, 2, 3, 4 and 5–10 km). The preflood and postflood changes (rate ratios) for each distance band to 5 km were controlled for the change in the 5–10 km band by estimating an interaction rate ratio (yielding estimates we refer to as ‘controlled pre–post changes’). Risk factors for antidepressant prescription that do not change over time are not confounders in this analysis, but we considered it possible that both neighbourhood population density and deprivation could be associated not just with prescription rates but also with *change* in prescription rates over time (prescription of antidepressants is rising sharply in the UK^18^) and so controlled for using these interaction terms. In addition, we also controlled for the number of prescriptions of all other (non-antidepressant) drugs as a log offset. CIs were adjusted for clustering by month using Huber-White estimators.^19^ Results were also stratified by flood type http://content.digital.nhs.uk/catalogue/PUB20664" and quintile of the neighbourhood deprivation scores. Trend in the pre–post change across the five flood distance bands was examined by fitting an interaction of the postflood indicator with distance score, rescaled to 0 for the furthest (5–10 km) and 1 for the closest distance band, yielding a smoothed contrast in the controlled pre–post change across bands.

Data linkage of practices, the flood database and socioeconomic deprivation markers were performed with ArcGIS V.10.3 and all statistical analyses with Stata V.14.

## Results

**Figure 1:**
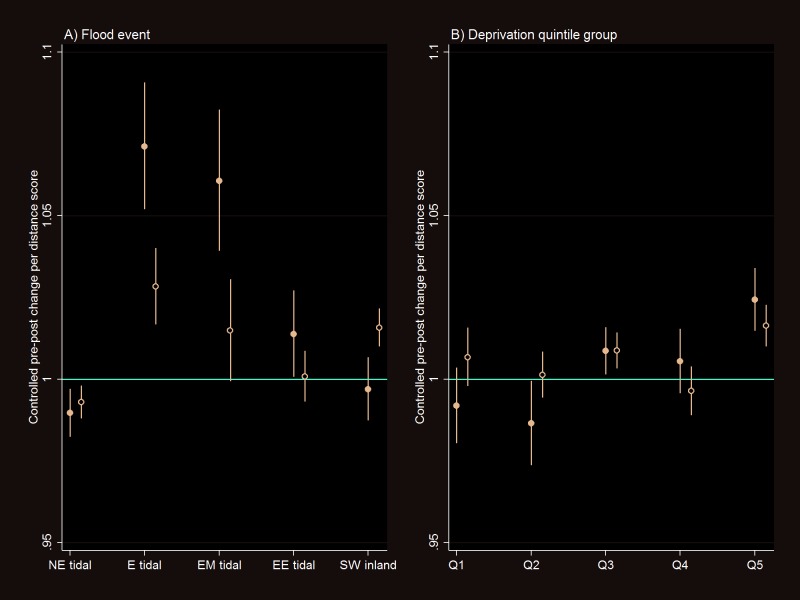
Controlled preflood and postflood change (interaction rate ratio) in the prescribed antidepressant drugs per distance score (scaled 0 for the furthest and 1 for the nearest distance band) by (A) flood event and (B) socioeconomic deprivation quintile groups (Q1 for the least and Q5 for the most deprived) for the major floods in England, 2011–2014. Filled circle shows that adjusted for year–month and impact of deprivation and population density on change in prescriptions over time and open circle shows that additionally adjusted for the number of non-antidepressant drugs. NE, North East; E, East; EM, East Midland; EE, East of England; SW, South West.

Nine hundred and thirty primary care practices were located within 10 km of the boundary of one of the selected five major flood events in England, 2011–2014 (see etable 1 in the online supplementary material). The number of dispensed antidepressant drugs prescribed by these practices was 9.7% greater in the postflood compared with the preflood year (table 1); the corresponding increase in non-antidepressant drugs was 4.1% (data not shown). However, even the most distant (5–10 km) band showed a 9.2% change in antidepressant drugs. When the pre–post change in prescriptions was controlled for (divided by) that in the 5–10 km band, the postflood increase in antidepressant drug prescriptions in the inner bands remained elevated by 0.76% (95% CI 0.44 to 1.08) in band 1 and 0.86% (0.35 to 1.38) in band 2 (table 1). There was evidence that the pre–post change in prescriptions increased with decreasing distance from the flood areas as reflected by the interaction terms (results shown unadjusted, adjusted for deprivation-related and population density-related change in prescriptions over time and with additional control for the trend in non-antidepressant drugs—table 1). Figure 1 shows the controlled pre–post change in antidepressant drugs per distance score by different flood event and quintile groups of neighbourhood deprivation score.

**Table 1:** Change of the number of antidepressant drug items prescribed at primary care practices a year before and after the flood by distance to an inundated area for major flood events in England, 2011–2014

Distance band	Preflood, n	Postflood, n	Preflood and postflood change (increase), %	Preflood and postflood change (increase) relative to that in the 5–10 km band (95% CI) (‘controlled pre–post change’)*, %	% Increase in the controlled pre–post change in prescriptions of the antidepressant drugs per distance score† (95% CI) adjusted for:		
					Unadjusted (model A)	Impact of deprivation and population density on change in prescriptions over time (model B)	Model B with further adjustment for prescription of the non-antidepressant drugs (model C)
<1 km	1 749 657	1 925 043	10.02	0.76 (0.44 to 1.08)	0.92 (0.59 to 1.26)	0.59 (0.24 to 0.94)	0.57 (0.23 to 0.92)
1 km	1 217 380	1 340 817	10.14	0.86 (0.35 to 1.38)			
2 km	759 145	834 835	9.97	0.71 (−0.04 to 1.46)			
3 km	660 737	723 398	9.48	0.26 (−0.24 to 0.77)			
4 km	576 816	628 265	8.92	−0.03 (−0.78 to 0.28)			
5–10 km	1 833 708	2 002 334	9.20	0			
Total	6 797 443	7 454 692	9.67	–	–	–	–

*Pre–post change relative to the furthest distance band (5–10 km) after controlling for month-to-month variation.

†Distance score is scaled as 0 for the furthest and 1 for the nearest distance band.

## Discussion

This study suggests an increase in prescribed antidepressant drugs in the year after flooding in primary care practices close to recent major floods in England. Although a very small change in relative terms (0.4%–1.0% increase among practices within 1 km of a flood area over the change observed in the furthest distance band), only a very small proportion of the patients registered with such practices will have been flooded. As an indication, we estimate that only 0.5% of postcodes within 5 km of such practices (an *upper* estimate for the catchment area of each practice) fell within a flood area.

It is a key limitation of our analysis that we had only practice-level data which will have meant very considerable dilution of any observable effect of flooding on antidepressant prescriptions because flooded households make up a very small part of the overall registered populations of these practices. The absence of detailed information on catchment areas does not allow us to investigate further the distances between home address and the practice. As such, we cannot know the degree to which the observed change in prescriptions reflects a causal effect confined only to flooded households, but if it were almost entirely this, the relative increase in the flooded households would be substantial and would be very broadly consistent with the results of questionnaire surveys of flood victims which suggest relative risks for anxiety and/or depression in the range of a twofold to fivefold increase.^4^ Another source of uncertainty (a probable conservative bias) is possible exposure misclassification from displacement of people or of practices themselves following a flood, which *might* in some circumstances affect recorded prescriptions data.

Nonetheless, against these limitations is the fact that using routine prescriptions data, although at very crude spatial resolution, provides a basis for objective analysis of change in antidepressant drug use among population partly affected by flooding. While it is not possible to eliminate all other factors that might be associated with change in prescription over time and space, our analyses controlled for (compared with) antidepressant prescription in preflood period, in other areas more distant from the flood, and non-antidepressant prescriptions. These comparisons strengthen the case of a specific effect of flooding on antidepressant drug use and thus presumably of clinical mental disorder. This is important as the literature is dominated by studies in which postflood questionnaire data on mental status, possibly subject to responder bias, have been gathered without baseline reference or control for change in non-flood areas. Mental health impacts may be among the most important adverse effects of flooding, especially for vulnerable individuals, and linked not only to the trauma of the flooding but also to the damage to property, social disruption and sometimes appreciable financial loss.^20^ However, although our analyses are consistent with a causal link, the degree to which the increase in prescribed antidepressant drugs observed in this study is actually concentrated among those directly flooded can only be determined by more detailed linkage studies.

**What is already known on this subject**

- Emerging reports suggest that flooding has adverse impacts on mental well-being among those affected. However, evidence remained limited as the most previous studies are based on survey or self-reports without robust preflood records.

**What this study adds**

- Linkage of administrative data, national flood database and primary care practices prescription provided a systematic approach of controlled interrupted time-series design with rigorous control for preflood period, other areas far from the flooded boundaries, major areal characteristics such as deprivation and population density and non-antidepressant drugs.
- This study demonstrated an increase in prescribed antidepressant drugs in the year after flooding in primary care practices close to recent major floods in England.
- Despite small increase in prescribed antidepressant drugs in relative terms, high prevalence of mental disorders implied substantial mental health burdens after flooding, suggesting important implications for public health practices after disaster.
- More detailed linkage studies are required to detangle the contribution of actual individual flood exposure.
