# Supplementary material for: Mental health impacts of flooding: a controlled interrupted time series analysis of prescribing data in England
Source: J Epidemiol Community Health. 2017 Aug 31;71(10):970–3. doi: 10.1136/jech-2017-208899 (PMC5754859; doi:10.1136/jech-2017-208899)
Supplement: Supplementary file 1 [file jech-2017-208899supp001.pdf]

Online supplementary material

Mental Health Impact of Flooding: A Controlled Interrupted Time-series Analysis of Practices Prescribing Data in England.

Authors: Ai Milojevic, Ben Armstrong and Paul Wilkinson

Contents:

eList. BNF (British National Formulary) names for antidepressant drugs identified in this study.

eFigure 1. Major flood events in England, 2011-14. These were selected as the set of events with more than 500 flooded addresses from June 2011 to November 2014 in England. Numbers in parenthesis show inundated area in square kilometre and the number of flooded addresses.

eTable 1. Number (%) of practices by flood event, deprivation, population density and distance band from practice to the closest flooded boundary.

eList. BNF (British National Formulary) names for antidepressant drugs identified in this study.

Selective Serotonin Re-update Inhibitors (SSRIs):
Citalopram, Cipramil, Fluoxetine, Prozac, Paroxetine, Seroxat Lustral, Sertraline

Serotonin-Norepinephrine Reuptake Inhibitors (SNRIs):
Duloxetine, Cymbalta, Lentreve Efexor, Venlafaxine, Desvenlafaxine (Pristiq), Venlafaxine (Effexor XR)

Noradrenergic and Specific Serotonergic Antidepressants (NASSAs):
Mirtazapine, Zispin

Tri-Cyclic and related Antidepressants (TCAs):
Amitriptyline, Imipramine (Tofranil), Tryptizol Tofranil, Amoxapine, Clomipramine (Anafranil), Desipramine (Norpramin), Doxepin, Nortyptyline (Pamelor), Protiptyline (Vivactil), Trimipramine (Surmontil)

Tetra-Cyclic Antidepressant (TeCAs):
Maprotiline

Dopamine receptor uptake blocker:
Burpropion (Wellbutrin)

5-HT2 Receptor Antagonists:
Nefazodone, Trazodone

Mono-Amine Oxidase Inhibitors (MAOIs):
Phenelzine, Nardil Parmate, Tranylcypramine, Isocarboxazid (Marplan), Selegiline (Emsam), Tranylcypromine (Parnate)

Noradrenergic Antagonist:
Mirtazapine (Remeron)

The above list does not cover whole range of antidepressant drugs, but covers major ones.

eFigure 1. Major flood events in England, 2011-14. These were selected as the set of events with more than 500 flooded addresses from June 2011 to November 2014 in England. Numbers in parenthesis show inundated area in square kilometre and the number of flooded addresses.

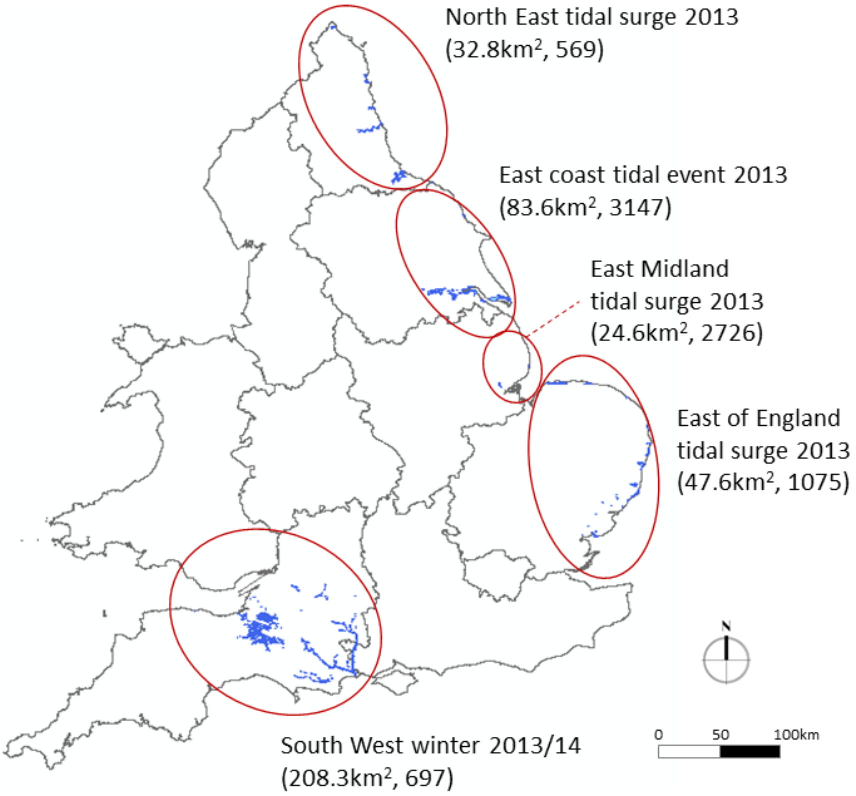

eTable 1. Number (%) of practices by flood event, deprivation, population density and distance band from practice to the closest flooded boundary.

	Distance band						Total
	<1km	1- km	2- km	3- km	4- km	5-10km	
All	220 (23.7)	157 (16.9)	105 (11.3)	86 (9.3)	89 (9.6)	273 (29.4)	930 (100.0)
Flood event							
North East tidal surge	66 (21.8)	63 (20.1)	30 (9.9)	31 (10.2)	25 (8.3)	88 (29.0)	303 (100.0)
East coast tidal surge	25 (21.4)	15 (12.9)	11 (9.4)	9 (7.7)	23 (19.7)	34 (29.1)	117 (100.0)
East Midland tidal surge	10 (32.3)	1 (3.2)	3 (9.7)	2 (6.5)	2 (6.5)	13 (41.9)	31 (100.0)
East of England tidal surge	29 (21.6)	16 (11.9)	15 (11.2)	7 (5.2)	11 (8.2)	56 (41.8)	134 (100.0)
South West winter 2013/14	90 (26.1)	157 (16.9)	105 (11.3)	85 (9.3)	89 (9.6)	273 (29.4)	345 (100.0)
Deprivation group [a]							
Q1 (the least deprived)	44 (23.7)	24 (12.9)	20 (10.8)	20 (10.8)	21 (11.3)	57 (30.7)	186 (100.0)
Q2	37 (19.9)	32 (17.2)	24 (12.9)	18 (9.7)	20 (10.75)	55 (29.6)	186 (100.0)
Q3	48 (26.0)	24 (13.0)	21 (11.4)	18 (9.7)	14 (7.6)	60 (32.4)	185 (100.0)
Q4	37 (19.8)	41 (21.9)	23 (12.3)	14 (7.5)	6 (3.2)	66 (35.3)	187 (100.0)
Q5 (the most deprived)	54 (29.0)	36 (19.4)	17 (9.1)	16 (8.6)	28 (15.1)	35 (18.8)	186 (100.0)
Population density group [b]							
Q1 (the least density)	47 (25.3)	20 (10.8)	15 (8.1)	18 (9.7)	17 (9.1)	69 (37.1)	186 (100.0)
Q2	43 (23.1)	27 (14.5)	19 (10.2)	20 (10.8)	13 (7.0)	64 (34.4)	186 (100.0)
Q3	24 (12.9)	20 (10.8)	25 (13.4)	21 (11.3)	25 (13.4)	71 (38.2)	186 (100.0)
Q4	41 (22.0)	49 (26.3)	16 (8.6)	17 (9.1)	26 (14.0)	37 (19.9)	186 (100.0)
Q5 (the highest density)	65 (35.0)	41 (22.0)	30 (16.1)	10 (5.4)	8 (4.3)	32 (17.2)	186 (100.0)

[a] Deprivation quintile groups defined by income and employment domains in IMD 2015 at LSOA level. Q1 for the least deprived and Q5 for the most deprived group.
[b] Quintile groups of the averaged population density across Output Areas within 2km of the practice. Q1 for the least density and Q5 for the highest density group.
